# Exon nomenclature and classification of transcripts database (ENACTdb): a resource for analyzing alternative splicing mediated proteome diversity

**DOI:** 10.1093/bioadv/vbae157

**Published:** 2024-10-29

**Authors:** Paras Verma, Deeksha Thakur, Shashi B Pandit

**Affiliations:** Department of Biological Sciences, Indian Institute of Science Education and Research (IISER)—Mohali, Punjab, 140306, India; Department of Biological Sciences, Indian Institute of Science Education and Research (IISER)—Mohali, Punjab, 140306, India; Department of Biological Sciences, Indian Institute of Science Education and Research (IISER)—Mohali, Punjab, 140306, India

## Abstract

**Motivation:**

Gene transcripts are distinguished by the composition of their exons, and this different exon composition may contribute to advancing proteome complexity. Despite the availability of alternative splicing information documented in various databases, a ready association of exonic variations to the protein sequence remains a mammoth task.

**Results:**

To associate exonic variation(s) with the protein systematically, we designed the Exon Nomenclature and Classification of Transcripts (ENACT) framework for uniquely annotating exons that tracks their loci in gene architecture context with encapsulating variations in splice site(s) and amino acid coding status. After ENACT annotation, predicted protein features (secondary structure/disorder/Pfam domains) are mapped to exon attributes. Thus, ENACTdb provides trackable exonic variation(s) association to isoform(s) and protein features, enabling the assessment of functional variation due to changes in exon composition. Such analyses can be readily performed through multiple views supported by the server. The exon-centric visualizations of ENACT annotated isoforms could provide insights on the functional repertoire of genes due to alternative splicing and its related processes and can serve as an important resource for the research community.

**Availability and implementation:**

The database is publicly available at https://www.iscbglab.in/enactdb/. It contains protein-coding genes and isoforms for *Caenorhabditis elegans*, *Drosophila melanogaster*, *Danio rerio*, *Mus musculus*, and *Homo sapiens*.

## 1 Introduction

Alternative Splicing (AS) process exerts transcriptome and proteome diversity in eukaryotic genes through generating variably spliced mRNA transcripts, which are translated into varied isoforms and may increase functional repertoire of a gene (Johnson et al. 2003, Light and Elofsson 2013, Modrek and Lee 2002). The orchestration of splicing is described through the following events (Kan et al. 2002): (i) exon skipping, (ii) mutually exclusive events, (iii) alternate 5' (5ss) or/and 3' (3ss) splice site, and (iv) intron retention (IR). Additionally, processes such as alternate transcription initiation/termination and alternate translational initiation/termination (ATI/ATT) also affect the transcript(s)/isoform(s). Although AS events are primarily described for coding exons, these could also involve untranslated regions or partially coding regions (Tapial et al. 2017, Leppek et al. 2018), where changes in the untranslated RNA secondary structure could potentially influence the translation rate, efficiency, and its stability (Tamarkin-Ben-Harush et al. 2017). The extent of AS is realized from multiple previous studies where it has been shown that ∼95% of multi-exon human genes are alternatively spliced into distinct isoforms (Pan et al. 2008, Barbosa-Morais et al. 2012, Li et al. 2016). Splicing plays an essential role in regulating various cellular processes such as transcription, apoptosis, autophagy, differentiation, and developmental processes (Wang et al. 2008, Wang and Burge 2008, Tang et al. 2013), and its aberrant regulation could lead to diseases.

The knowledge of association between exonic variations (including indels) and isoform(s) would facilitate understanding the roles of AS in ATI/ATT, altering protein sequences (such as translational frameshift, truncation), and function. Such details are embedded in primary databases documenting well-annotated alternatively spliced gene transcript(s)/isoform(s), for instance, NCBI (O’Leary et al. 2016), Ensembl (Cunningham et al. 2022), and UCSC genome browser (Lee et al. 2022). However, tracking exons or their splice/sequence variations in gene architectural context is cumbersome and mostly non-trivial. There have been limited studies on characterizing exons for such features. For example, ASPicDB documents multi-exon gene protein variants with their various predicted properties (Martelli et al. 2011). ASTRA and ASTALAVISTA used concepts of naming bit matrices to define AS, ATI, and ATT events followed by their conversion to a decimal system or symbolic event designation (Nagasaki et al. 2006, Foissac and Sammeth, 2007, Sammeth et al., 2008). These approaches primarily derive exon characteristics from pairwise comparisons of transcripts identifying local splice-altering events.

We developed an exon-centric framework, Exon Nomenclature and Classification of Transcripts (ENACT), to circumvent challenges in exon characterization (Verma et al. 2024). This framework allows the tracking of exon(s) and their known variants derived from transcript models in NCBI RefSeq (O’Leary et al. 2016). The relative position of exon(s) and its splice variants are determined using their genomic coordinates (GC) and exon(s) coding status obtained from Coding Genomic Coordinates (CGC). Cumulatively, these attributes are encoded in a six-character alphanumeric exon unique identifier (EUID) assigned uniquely to exon variants (Verma et al. 2024). This systematic and comprehensive annotation of exons is amenable to global tracking of features necessary to depict the exonic entity’s role in gene architecture. Subsequently, we have associated predicted features at the level of exons for each isoform to enrich their annotations, which facilitates unraveling their functional variability.

We annotated exons/transcripts of genes encoded in genomes of five model organisms and documented them in the ENACT database (ENACTdb). The database server provides user-friendly visualization of isoforms represented as a combination(s) of exons with their associated features to aid in efficient analysis for interpreting exonic variations’ impact on isoform variants. The visualization utilizes the state-of-the-art Nightingale library for simultaneous display of exon entity-based transcript representations with their predicted protein features. Thus, enhancing isoform feature depiction while retaining exonic information. The comprehensive and exon-centered visual tool available in the ENACTdb will help the scientific community to explore, gain insights, or decipher detailed associations of splicing exonic variations linked with intragenic isoform variability.

## 2 Availability and implementation

### 2.1 Description of transcript/isoform ENACT annotation

**Figure 1.**
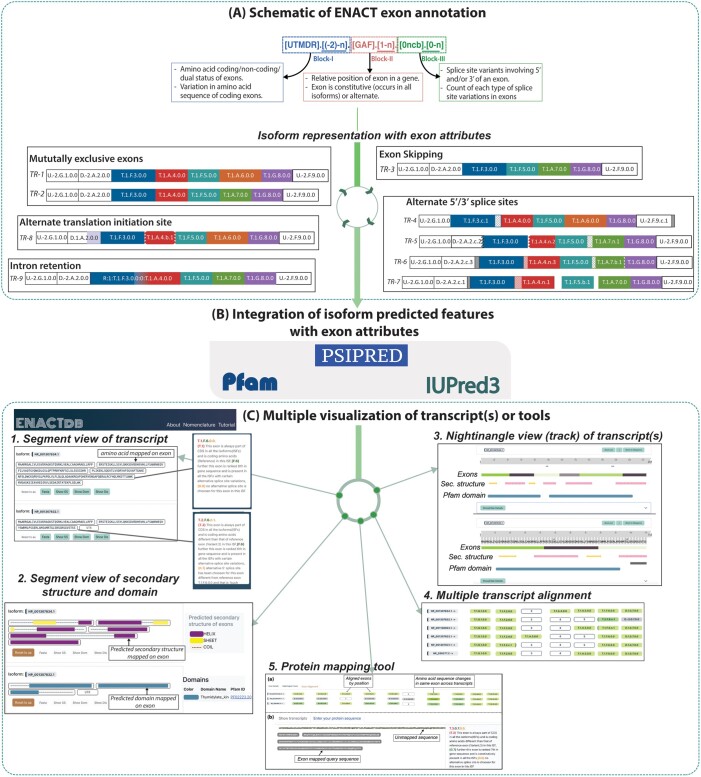
Hierarchical flowchart of the ENACTdb from nomenclature to visualizations. (A) shows ENACT nomenclature and annotation of transcripts/isoforms of a hypothetical gene using exon annotation and highlights various splicing events. (B) illustrates integration of isoform prediction at the level of exons, and (C) depicts various visualization views available with ENACTdb web server and protein mapping tool.

The exons of a gene are annotated using the ENACT framework (summarized in Fig. 1A), which encapsulates the following exon features: amino acid coding status (Block-I), relative position with exon occurrence frequency (Block-II), and splice site variations (Block-III). We extracted the transcript/isoform details along with GCs and CGC of all exons of a gene from NCBI RefSeq database (Supplementary Fig. S1). A reference isoform with the maximum number of coding exons is selected to construct an initial reference set of exons (RSOEx). This set is expanded to include exons from other isoforms that do not intersect the GCs of RSOEx. Thus, obtained non-overlapping exons of RSOEx are numerically sorted to allocate them relative positions in the gene architectural context. The remaining exons overlapping with the reference set are defined as splice site relatives or are classified as IR events when exon GCs overlap with two or more exons/variants (Verma et al. 2024). Subsequently, we probe the amino acid contribution of exons (using CGC) within a transcript/isoform to characterize their coding potential (Supplementary Fig. S1). Since an exon can be part of the coding sequence in one transcript but not in others, its coding status is tracked both for individual transcripts and cumulatively across all transcripts. As mentioned above, six alphanumeric characters of EUID characterize an exon (Supplementary Fig. S1). Their detailed notation is discussed in Supplementary material (Supplementary S1 Text) and described in (Verma et al. 2024). We grouped EUID into three blocks (Fig. 1A) to easily interpret their encoded features. Since IR events involve two or more exons, it is annotated as a combination of their EUIDs as described in supplementary material (S1 Text).

### 2.2 Description of ENACTdb

Transcripts tables for protein-coding genes of five representative model organisms, viz. *Caenorhabditis elegans, Drosophila melanogaster, Danio rerio, Mus musculus*, and *Homo sapiens*, were obtained from NCBI RefSeq and annotated exons using the ENACT framework. Additionally, protein features such as secondary structure (PSIPred) (Jones 1999), Pfam domains (Mistry et al. 2021), and disordered region (IUPRed3) (Erdős et al. 2021) were predicted (Fig. 1B) and mapped to exons (Supplementary S1 Text). This framework allows tracking of exon(s) and their known variants as alternatively spliced isoforms from transcript models specified in NCBI RefSeq (O'Leary et al. 2016). These are documented in ENACTdb, which is integrated with an interface for easy retrieval and a user-friendly display to represent transcript/isoform information for interpretations. The database schema of various exon/transcript/isoform features and their associations with each other are depicted in the schema (Supplementary Fig. S2). The visualization interface is rendered through ReactJS, and Django supports backend. ReactJS enables single-page design, so users can conveniently analyze the exon properties of transcripts. Additionally, ReactJS state-based containment of transcripts enables the selection of more than one transcript (with their independent controls) to compare them and view switching of listed transcripts without losing their sequential order. The database offers a user-friendly visual exon-centric representation of isoform that shows AS events with protein features facilitating the exploration of exon variation impact on intra-gene isoform diversity.

## 3 Features of ENACTdb web server

ENACTdb offers an interface to retrieve a gene and its associated detailed isoform annotations for visual interpretation. A user can query using a gene name or NCBI gene identifier, resulting in a gene list along with its source organism (Supplementary Fig. S3). After selecting a gene from the search output, a user can explore its annotated features in representations (views) discussed below. Accessing the main page (by clicking “ENACTdb,” enabled on all pages) will initiate a new search. Subsequent to the selection of gene of interest, the relevant details are displayed in two sections. The top section shows general gene properties, such as transcript counts and its exon (coding/non-coding) composition. The lower section has a scrollable header listing all isoforms (cross-referenced to NCBI) with an option to show/hide various isoform representations in the display box below it. Additionally, it also contains protein length, exon count, and predicted fraction of secondary structure for each isoform (Supplementary Fig. S4).

### 3.1 Transcript views

The isoform and its constituent exon properties are rendered in two switchable transcript views, viz. Segment and Nightingale, each supporting interactive features for practical visual analyses. These differ in their exon depiction, where the Segment view shows exons as rectangular containers with options to display various mapped features. In contrast, the nightingale view offers simultaneous feature components in modular sections. We used the human deoxythymidylate kinase (*DTYMK*) gene to illustrate the functionality of ENACTdb for visual representation and interpretation (Fig. 1).

#### 3.1.1 Segment view

Its default view displays the chosen isoform of a gene as a linear arrangement of constituent exons represented as a rectangular box with amino acid sequence overlaid on it (Fig. 1C1). The hovering over the exon readily shows its EUID (detailed exonic attributes) in a right panel window, facilitating visual interpretation of its features (Fig. 1C1). The selection of an appropriate property can switch over the display of predicted sequence features mapped on the exon. “Show SS,” “Show Dom,” and “Show Dis” display secondary structures, Pfam domains, and disordered regions, respectively (Fig. 1C2). A user can display multiple isoforms to compare exonic or other predicted features across them. As shown in Fig. 1C2, isoforms NP_001307832.1 and NP_001307834.1 of the DTYMK gene share four of six exons. However, the former has a truncated sixth exon (variable 5'ss), while the seventh is non-coding, unlike the latter. On selecting the domain view, it is apparent that NP_001307832.1 (IS-6) has only one occurrence of Pfam domain Thymidylate_kin, while the IS-2 (NP_001307834.1) has two domains (Supplementary Fig. S5).

#### 3.1.2 Nightingale (ProtVista) view

Using ProtVista (Watkins et al. 2017) and its successor Nightingale project (Salazar et al. 2023), we implemented their protein viewing modules with the necessary modifications of the script/code for the simultaneous display of exon, isoforms overlaid with predicted features (Fig. 1C3). A slider (zoom button) is provided for seamless zooming in/out to the level of protein sequence, which concomitantly shows sequence, exons, and predicted features (Fig. 1C3) arranged in horizontal rows each for exons, secondary structure, disordered regions, and Pfam domains. The mapped exons are shown in alternate colors to distinguish exons from their immediate neighbors. This reviewing pane is accompanied by a data entry table, which provides data associated with rendered features. Additionally, there is a synchronization between data entry table items and reviewing pane, where clicking on either highlights relevant entry/view.

#### 3.1.3 Transcript alignment view

As inferences of exonic variations can be derived by reviewing their occurrences across transcripts, we provide a unique feature in ENACTdb to display multiple transcript alignments based on exon-relative positions. These could provide a perspective of exonic combination effects on sequence, frameshifts, and co-occurrence of exons across transcripts. For instance, the influence of variable occurrences of exons 3–5 in the *DTYMK* gene can be readily observed in multiple transcript alignment (Fig. 1C4). Accordingly, changes in the predicted features due to exonic variations can be viewed and analyzed in the nightingale view.

#### 3.1.4 Mapping exons to a protein sequence

An exon-mapping tool is developed to identify the occurrence of known exons in a user-provided input protein sequence. The method relies on string matching of sequences (exon to input), making it essential to know the source gene of the input sequence. After running the tool, the matching sequence is output as a mapped exon, and the unmatched sequence is left as indicated in the input sequence. We illustrate this tool by submitting an N-terminal-modified PTEN isoform variant, which showed match to four exons in the output, where, as expected, the N-terminal-modified sequence remains unmapped (Fig. 1 (C5)).

The transcript annotations of ENACTdb genomes are coordinated with genome assembly/patch updates in NCBI RefSeq. In future release of our database, we will include exon/transcript annotations from other representative genomes (NCBI RefSeq).

## 4 Discussion

ENACTdb implements a unique framework to annotate exons with their variations, and predicted protein attributes for protein-coding genes of five model organisms. These annotations enable an intuitive insight into how exonic variations affect transcript or protein features. The modular components of Django and ReactJS provide user-friendly interactive interface for visualization and straightforward interpretation of exons in transcripts. The ENACTdb data will be updated regularly with genome assembly/patch updates in the NCBI RefSeq database. Collectively, ENACTdb will assist experimental and computational biologists in deciphering details of AS and associated processes in evolution.

## Supplementary Material

vbae157_Supplementary_Data

## Data Availability

The data underlying this work are available at https://www.iscbglab.in/enactdb.
