# Supplementary material for: Exon nomenclature and classification of transcripts database (ENACTdb): a resource for analyzing alternative splicing mediated proteome diversity
Source: Bioinform Adv. 2024 Oct 29;4(1):vbae157. doi: 10.1093/bioadv/vbae157 (PMC11576355; doi:10.1093/bioadv/vbae157)
Supplement: vbae157_Supplementary_Data [file vbae157_supplementary_data.pdf]

Supplementary information for the article titled

"Exon Nomenclature And Classification of Transcripts database (ENACTdb): A resource for analyzing alternative splicing mediated proteome diversity"

Paras Verma, Deeksha Thakur, Shashi B. Pandit*

*To whom correspondence should be addressed

email: shashibp@iisermohali.ac.in

Affiliations

Department of Biological Sciences, Indian Institute of Science Education and Research (IISER) – Mohali, Knowledge City, Sector-81, SAS Nagar, Manauli PO 140306, India.

S1 Text: Detailed features of exon nomenclature

The unique descriptor or Exon Unique IDentifier (EUID) in ENACT comprises six characters, each encompassing its one distinct feature delimited by a dot ('.'). The detailed characteristics and information ingrained in these characters with their notation are discussed in Verma et al.[1], and briefly discussed below:

A. Exon amino acid coding attribute: The whole or partial region of an exon (genomic coordinate) can encode an amino acid sequence in one or many transcripts, or it can be non-coding in transcripts wherever it occurs. We use alphabet notation to describe an exon's amino acid coding property across transcripts. These are defined as follows:

- T: It depicts that an exon (or its splice site variants) always codes for amino acid sequence or is a part of coding sequence (CDS) in a transcript. The exon region has a defined Coding Genomic Coordinate (CGC) in all transcripts.
- U: It shows that an exon (or its splice site variants) forms the untranslated region of transcripts whenever it occurs or does not have CGC.
- D: It shows that an exon (or its splice site variants) is part of CDS in at least one transcript and is part of UTR in another transcript(s). Notably, these exons should share identical Genomic Coordinates (GC) across transcripts existing as either coding or non-coding.
- M: It is assigned for single nucleotide protein coding exon in the CGC region.
- R: It is for an intron retention exon, described later in the nomenclature section.

B. Exon amino acid sequence variation: Exons with the same GCs in different transcripts might yield distinct amino acid sequences because of alternate promoter sites or ATI/ATT events. In addition to these factors, the splice site change in the upstream exon(s) could induce a shift in the reading frame, also described as frame-shift events (FSE), resulting in an altered amino acid sequence of an exon. These changes are described in the nomenclature by a numeric character with the following definitions:

- -2: It denotes that the exon contributes no amino acid sequence.
- 0: It is a primary placeholder to depict the M case (single nucleotide exons)
- -1: It depicts premature stop codon in the upstream exon; hence, this exon does not contribute to the amino acid sequence, even though it has more than one nucleotide in the CGC.
- 1: It shows that the exon contributes amino acids to the isoform.
- ≥2: It is a counter denoting the number of different amino acid sequence variants observed for an exon with the same GCs.

C. Exon occurrence frequency: We computed the Weighted Inclusion Frequency (WIF) of exons as a ratio of exon occurrence in transcripts by the total number of transcripts. The WIF is discretized into groups with the following notation:

- G: Exons that have a WIF of 1 are classified as constitutive exons.
- A: Exons having WIF < 1 are classified as alternate exons
- F: It refers to exons in all transcripts but has 5ss and/or 3ss splice site variations. The 'F' tags are also called constitutive-like exons.

In particular, the 'G' code is assigned to all exons in a gene that has a single transcript.

D. Exon relative position in a gene: The exons in RSOEx are numerically sorted based on the GC position. These are assigned relative positions from one to N (total number of exons in RSOEx) in a gene.

E. Alternate splice sites: RSOEx entities can have differing splice variant instances (5' and / or 3' exon) in other transcripts, which are inferred after comparing their GCs. The notations of splice sites are defined as follows:

- n: It denotes the change only in GC of the exon 5' splice site with no change in the 3' GC of representative exon.
- c: It shows variation only in GC of exon 3' splice site with unchanged 5' splice site GC of representative exon.

- b: It is used when 3' and 5' splice sites of an exon vary in GC with respect to representative exon.
- 0: It is the default value used for anchor exons.

Any of the above variations could either extend or shorten the length of an exon.

F. Occurrence of splice site changes: The last character of the ENACT descriptor is a numeric digit, which is a splice site change counter to keep track of the number of these events observed for an exon in a gene. The default exon count starts from 0 and is used for anchor exons. The counter is incremented by one with observation of every n/c/b type of variation. The number of n/c/b events of an exon in a gene can be obtained by looking at the total instances of 5ss or/and 3ss.

Features of three blocks in EUID are briefly described below:

a. Amino acid coding status in transcripts such as coding/non-coding or both. (Block-I)
b. Contribution of amino acid to the transcript/isoform. (Block-I)
c. Inclusion frequency of exon in transcripts. It is categorized as constitutive (occurs in all transcripts), constitutive-like (occurs in all transcripts with splice site variations) and alternate. (Block-II)
d. Relative position of exon in the gene. (Block-II)
e. Type of splice site variations (5'ss and/or 3'ss) of an exon. (Block-III)
f. Numeric count of splice site variations observed in an exon. (Block-III)

Intron Retention (IR): It is a special case of exon nomenclature where the six-character descriptor is insufficient to capture details of an intron retention event. To describe this, we use five separate identifiers combined with the ':' colon symbol to distinguish from the standard EUID. The first identifier is the alphabet 'R' to recognize that the exon is involved in IR, followed by a digit describing its amino acid coding attribute (the same notation is used as described before). The third and fifth identifiers are exon EUIDs, between which the intron/exon region is retained to form the IR exon. The fourth identifier is a numeric character showing the number of retention events observed involving exons and their variants. We use 0 as the default value of this counter. For instance, a IR event can be depicted as:

R:1:T.1.A.4.c.1:0:T.1.A.5.c.1

The above identifier indicates IR event as amino acid coding, with retention of intron region between exons 1.1.A.**4**.c.1 to T.1.A.**5**.c.1, while being first IR instance involving variants of exons 4 and 5 (shown in bold as these are the relative positions of exons in a gene).

Pfam domains (v35.0) were predicted for each isoform using PfamScan (HMMERv3.2.1) with e-value cut-off as 0.01. The secondary structure prediction was performed using modified PSIPred paraments from I-TASSER package. The disorder region was predicted using IUPred3 where a score >0.5 was used to predict residue-level disorder.

S2 Text: ENACTdb database design and schema

The exon nomenclature and gene annotations of 5 model organisms obtained from NCBI are documented in ENACTdb. We have designed and implemented exons/transcripts/genes information in MySQL tables to manage multiple exon features. The Figure S1 shows the database schema, which illustrates 3 primary tables, called exonapp_genes, exonapp_transcripts, and exonapp_exons. Exons are kept in separate tables because it reduces redundancy, as the same exon can be present in multiple transcripts. In general, the table _genes view is a collection of all genes in an organism, the _transcripts table has a list of transcripts associated with a gene, and all exons in a gene are listed in the _exons table. Such a cross-reference of tables allows one to reconstruct transcripts with exons easily for any given gene. The secondary tables contain exon entries (unique properties are rows) associated with a list of transcripts. Although there is an increase in manual mapping and transcript construction, it is optimized for space. ReactJS renders the front-end GUI and is a one-page application (https://reactjs.org) (Virtual DOM works by turning on/off different components and generating content dynamically) that renders the front page. The Django server manages the backend view of SQL tables, other web server management tasks, and fetches the information to the front-end server.

The gene encoded in genomes of model organisms *Caenorhabditis elegans* (worm), *Drosophila melanogaster* (fruit fly), *Danio rerio* (zebrafish), *Mus musculus* (mouse), and *Homo sapiens* (human) were obtained from NCBI and processed by Enact algorithm to assign exon nomenclature.

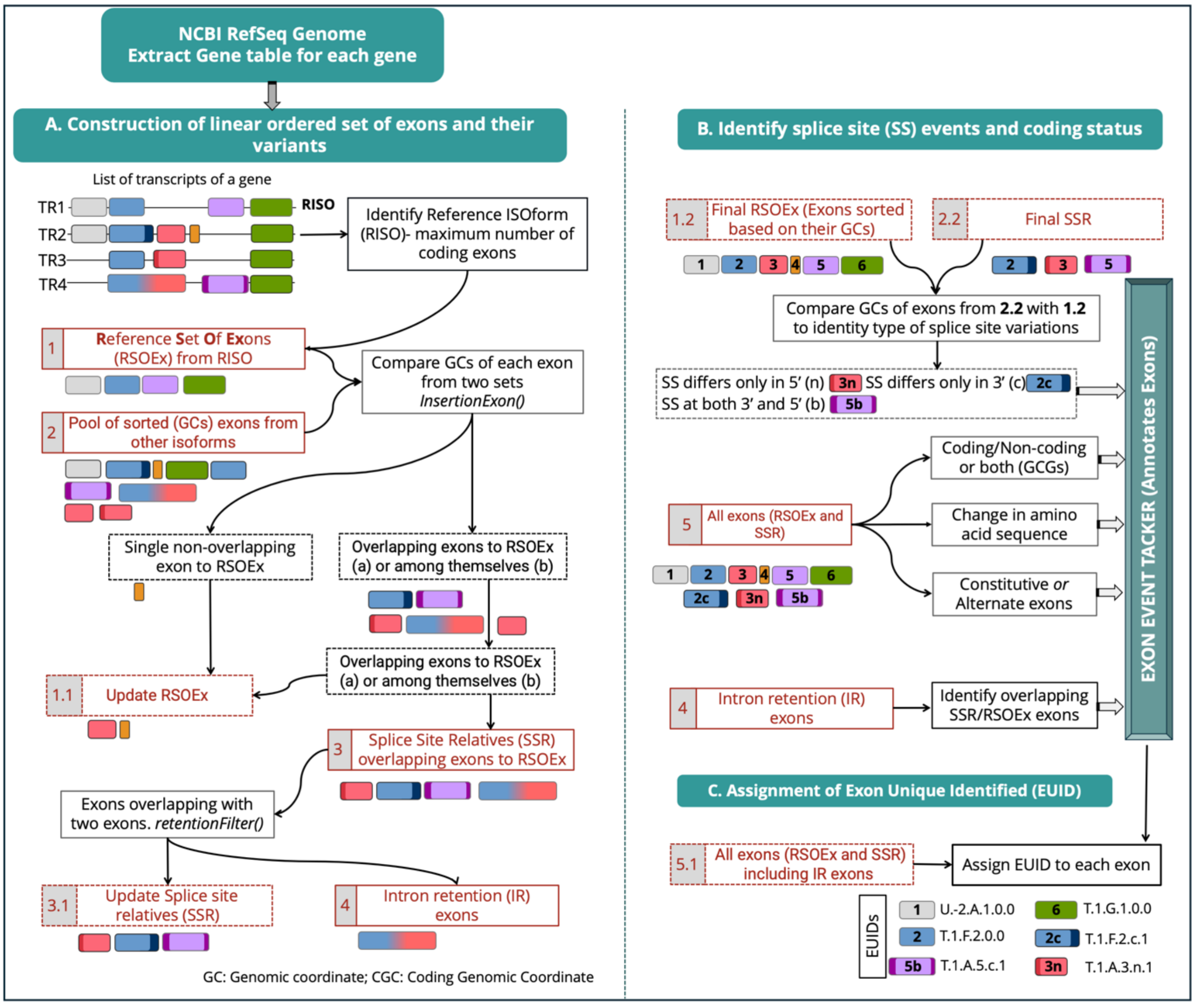

Figure S1: **Overview of ENACT algorithm of exon annotation**. Panel (A) shows flowchart describing the algorithmic steps for constructing the Reference Isoform (RISO), identifying splice variants, and intron retention instances. Briefly, a reviewed transcript/isoform having the maximum number of coding exons is chosen as RISO. The exons of RISO constitute an initial set of reference exons (RSOEx), which is subsequently updated with non-overlapping exons from other transcripts (steps 1 and 1.1). Once the RSOEx set is established, the exons are sorted according to their genomic coordinates (Step 2). During this sorting process, exons from all transcripts that overlap with RSOEx are identified as splice site relatives (Step 3). Here, splice site variants are annotated with notations 'n', 'c', and 'b' referring to 5', 3', and 5' with 3' splice site change, respectively, in exon instances. Exons overlapping with two or more RSOEx entities are defined as intron retention cases (Step 4). Flowchart in Panel (B) describes the procedure for compiling exonic attributes obtained from previous steps with coding genomic coordinates and exon occurences in transcripts. Based on the above, attributes are designated to exonic loci (relative position of reference exons). An exon is defined as coding if it consists of coding genomic coordinates; otherwise, it is non-coding. Further, constitutive and alternate

notation of exon refers to its occurrence in all and some transcripts, respectively (step 5). Various splice variant notations are sourced from Step 3, and intron retention from Step 4. Lastly, Panel (C) illustrates the construction of Exon Unique IDentifier (EUID) from attributes compiled in previous steps (panel B). The main steps are indicated with solid red colored rectangular boxes are and sub-steps in dotted red colored rectangular boxes.

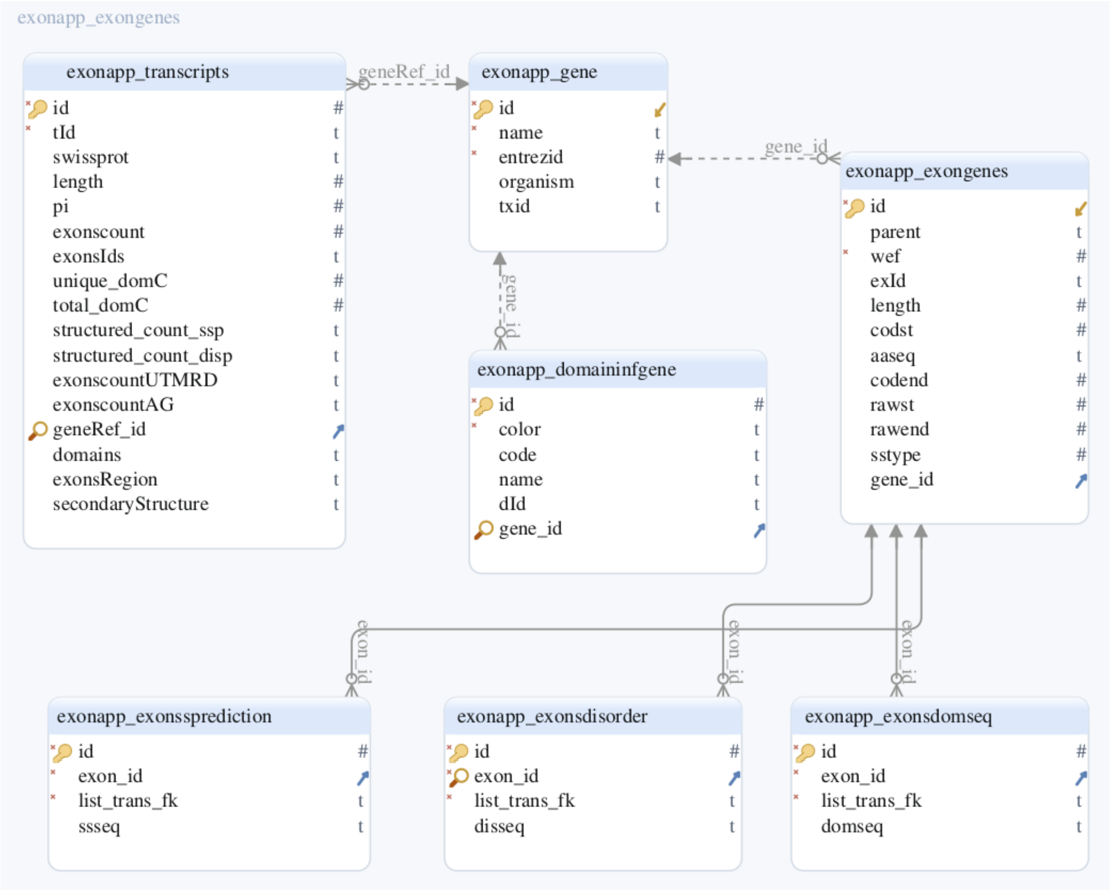

Figure S2: Database schema of ENACTdb. The figure shows the ENACTdb relational database SQL tables and their relationship between entities of the SQL tables.

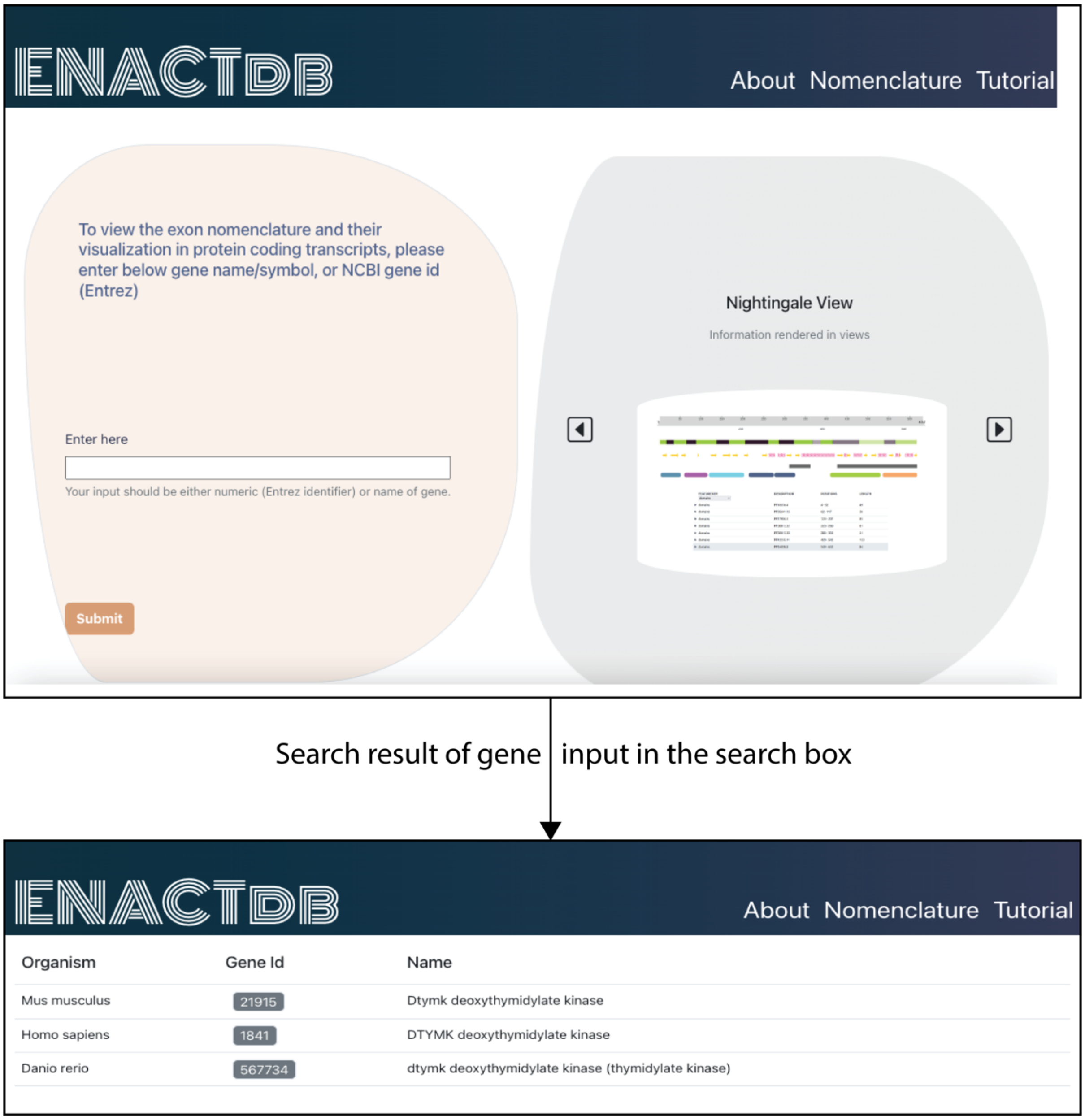

Figure S3: ENACTdb user interface for submitting a gene query and output of the same.

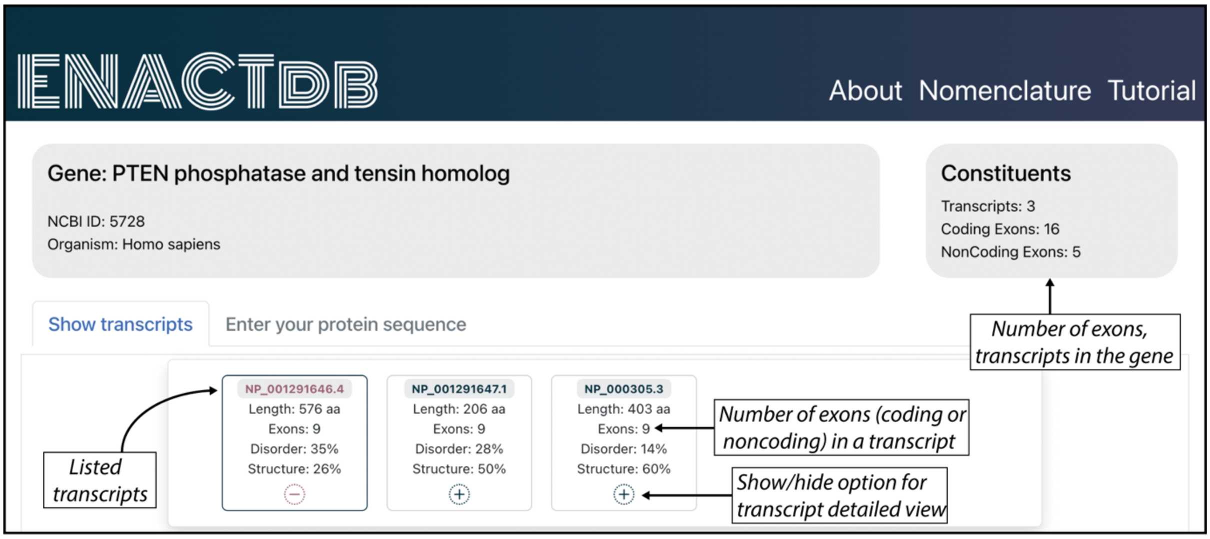

Figure S4: Gene view rendered by the ENACTdb server showing various embedded information on the page. The results are shown for hPTEN gene.

Annotated exon architecture in human genes

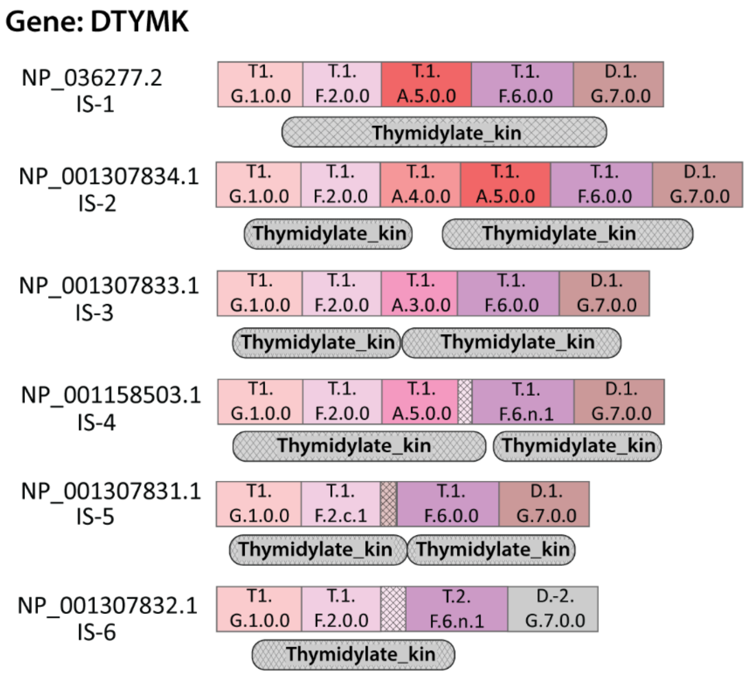

Figure S5: Detailed isoform annotated with ENACT exon nomenclature of all known isoforms of human DTYMK gene

References

1. Verma, P., D. Thakur, and S.B. Pandit, *Exon Nomenclature and Classification of Transcripts (ENACT): Systematic framework to annotate exon attributes.* bioRxiv, 2024: p. 2024.06.07.597685.
